# Multiple struggles in fighting violence against women: implications among Romani women leaders in Spain

**DOI:** 10.1080/16549716.2017.1317084

**Published:** 2017-06-06

**Authors:** Carmen Vives-Cases, Eva Espinar-Ruiz, Esther Castellanos-Torres, Anna-Britt Coe

**Affiliations:** ^a^ Department of Community Nursing, Preventive Medicine and Public Health and History of Science, Alicante University, Alicante, Spain; ^b^ CIBER of Epidemiology and Public Health, Barcelona, Spain; ^c^ Public Health Research Group, Alicante University, Alicante, Spain; ^d^ Department of Sociology II, Alicante University, Alicante, Spain; ^e^ Interuniversity Institute of Development and Peace, Alicante University, Alicante, Spain; ^f^ Epidemiology and Global Health Unit, Department of Public Health and Clinical Medicine, Umeå University, Umeå, Sweden

**Keywords:** Intimate partner violence, Roma population, gender inequalities, intersectionality, qualitative study, Gender and Health Inequality - intersections with other relevant axes of oppression

## Abstract

**Background**: Violence against women (VAW) is a central issue in gender studies and one that has united feminist activists from around the world. But this does not mean that this struggle is singular: indeed, one can say that there are many, diverse and sometimes even contradictory struggles occurring throughout the world.

**Objectives**: To identify and analyze the different struggles faced by women from Roma organizations in Spain in relation to VAW and their work with affected women.

**Methods:** Twelve semi-structured interviews were conducted among women actively involved in Roma associations in different Spanish cities, in 2015. An inductive thematic analysis was used to analyze the empirical materials.

**Results:** Our analysis resulted in three themes that captured different struggles that women from Roma organizations have faced: ‘between persistence and rupture of restrictive gender norms’, ‘invisibility and normalization of violence against women’ and ‘willingness and trust in daily work with women’. The activities related to VAW carried out by the interviewed women were more related to their personal initiatives and abilities than to strategies proposed by the organizations they work for.

**Conclusions:** The results show the need to support the initiatives of Romani women and their own struggles related to identity. This is what makes them true promoters of social change and, more specifically, change related to gender relations both within and outside of their communities.

## Background

Violence against women (VAW) is a central issue in gender studies and one that has united feminist activists from around the world. Women’s activism has been associated with significant changes in women’s knowledge and use of social and health services, as well as changes in social attitudes about gender, acceptance of this problem and increasing support for local efforts [1]. According to a recent literature review, VAW prevention programs should support collective and pro-feminist activism to promote a strong positive impact at both the community (enabling an environment for equality and non-violence) and individual (inspiring women and men to become agents of change) levels [2].

This paper focuses on the emergent work developed by Romani women in civil society groups. The Roma is the largest ethnic minority group in Europe with an estimated population of 10 million in the European Union (EU) and several million more in countries outside the EU [3]. It is characterized by a range of different languages, customs and means of ethnic self-identification. However, wherever they reside, Roma populations are more exposed to social exclusion processes than the general population are. These social exclusion processes negatively influence access to resources, social opportunities and the exercise of basic rights, resulting in poorer outcomes in terms of health and well-being [4,5]. Compared to the general population, the health of the Roma population is disproportionately affected by social determinants including low levels of school attendance and exposure to unsuitable living conditions [6]. This community presents higher rates of morbidity and mortality and worse self-perceived health, and it is more likely to experience functional disability and chronic health problems such as diabetes [7–9]. Prior studies show that the Roma community participates less in the labor market and education system, is more concentrated in urban areas with poor housing conditions and experiences discrimination [10]. It has also been observed that the health indicators for the Roma population are generally at worse levels than those of the most disadvantaged social class of the general population [11].

In less than a decade, a number of Romani women activists, mostly in Central and Eastern Europe, have brought to light issues of multiple forms of discrimination (based on race, socio-economic status, sexual identity and/or orientation and gender). Activists have also noted the systematic exclusion of Roma in accessing healthcare, employment, education and political representation. More controversial issues have also been highlighted, such as forced sterilization, early marriage, forced prostitution and VAW [12–15]. To do so, Romani women have had to face different struggles related to the intersection of different axes of discrimination such as sexism, racism and classism [4].

Romani women have more often been studied as patients or victims than as leaders of social change [16]. In the case of VAW research, Romani women have received little attention, although the few studies that do exist indicate alarming figures. A study conducted in Bosnia and Herzegovina in 2011 estimated that 43% of Roma women had experienced physical violence by their partners and that 36% had been abused psychologically [15]. In another study conducted in Turkey, it was estimated that Roma women have a three times greater risk of VAW than women in the general population [17].

Currently, there are only a few studies about how Romani women activists negotiate different, and sometimes contradictory, ethnic and gender identities, from a position that involves struggling against, comprehending, acknowledging and even sharing the social norms dominant in their contexts [18–21]. Describing these struggles is important for developing VAW responses to Romani victims as well as for enhancing the effectiveness of other sectors’ interventions related to VAW.

### The intersectional approach for the study of Romani women’s responses to VAW

This study used an intersectional approach as its theoretical framework [22,23]. Introduced by Black feminist scholars in the 1980s, an intersectional approach assumes that the social locations of groups and individuals are shaped by intersecting systems of power relations, where gender is one axis of inequality and discrimination, alongside class, age, race/ethnicity and sexual identity/orientation. The intersection of these axes creates positions of privilege and disadvantage, inclusion and exclusion, and these positions affect how individuals perceive themselves, how they are perceived and treated by others and how they interpret problems [22,24].

Romani women experiencing VAW are jointly exposed to at least three sources of oppression. They may be oppressed for being female, for being Roma and for being poor. In addition, the Roma community in Europe is composed of several sub-communities differentiated by settlement model, culture and religion, legal status, language and period of migration [4]. There are also differences within these groups based on the political and cultural context of their place of residence. These differences, in addition to the intersecting systems of power relations (gender, ethnicity, class and/or sexual orientation), interact to create specific forms of subordination. The consequences can include reduced access to information about institutional VAW-related resources, limited trust in professionals due to discrimination, difficulty discussing intimate relationship problems with strangers (especially if they are men), language or cultural issues, shame associated with being a victim of VAW and/or fear of losing children, among other issues [25,26].

The intersectional approach has also been previously applied to show how Romani activists usually have to face many challenges related to their position vis-a-vis the general discourse of the Romani movement and the general feminist discourse [27] and the controversial relationships across different generations. These challenges are present in their daily work as cultural mediators in relation to different health issues [28,29].

The main aim of our study is to identify and analyze the different struggles that women from Roma organizations in Spain face in relation to VAW and their work with affected women. We considered the intersectional approach as the best approach for achieving this objective.

## Methods

A qualitative study based on 12 in-depth interviews with Romani women who were actively involved in Roma and pro-Roma Spanish associations was performed in 2015 in different cities in Spain. This study was part of a bigger project that aimed to analyze primary health services’ and professionals’ readiness to manage violence against Romani women in Spain. The larger project also aimed to propose strategies to improve responses in these cases, taking into account the health professionals’ and Romani women activists’ perspectives.

Purposive sampling was carried out for the whole project sample, including participants who were involved in different types of associations (pro-Roma but not exclusively Roma NGOs, Roma organizations, Romani women’s groups and feminist groups of Romani women). The first strategy employed to recruit participants was to contact a Roma association in Alicante – where part of the research team is located – and other relevant associations. These associations were identified through an initial Internet search for Roma organizations that had promoted VAW interventions or programs involving health professionals. In addition, we employed the snowball method – in each interview, information was requested concerning possible contacts. A total of 28 organizations were contacted, and 12 agreed to participate. Associations were contracted to select interviewees, given their experience working in VAW-related interventions.

The interview guide consisted of a few topics for discussion without a predetermined sequential order. The topics were related to perceptions about VAW and existing responses in Spain in general, and in relation to primary health services in particular. Topics were also related to interventions and programs developed within the associations, and factors surrounding the effectiveness of these program activities. Other themes related to interviewees’ experiences as Romani women actively involved in Roma associations. The topic of changes in Romani women’s position emerged during the interview, although it was not initially addressed in the guide (Table 1).

**Table 1.** Interview guide addressed to women from Roma or pro-Roma associations in Spain, 2014–2015.

As a part of the work of your association, do you carry out any activities related to intimate partner violence in the Roma population?
(IF YES) Can you tell me about these activities?
In your opinion, are these programs directed towards intimate partner violence in the Roma population effective? Why?
(IF NO) Are you aware of, or have you participated in any activity outside of your association related to intimate partner violence in the Roma population?
In your opinion, are these programs directed towards intimate partner violence in the Roma population effective? Why?
Is there a collaborative relationship between the administration and your association that supports intervention related to intimate partner violence with Roma women?
(IF YES) How would you describe this collaborative relationship (administration/association)?
Does your association attend to victims of abuse?
(IF YES) Do you know if the Roma women who suffer abuse and who attend your association also attend a healthcare center after having suffered an attack? Why?
(IF NO) In your personal experience, do you believe that Roma women who are victims of abuse attend a healthcare center after having suffered an attack? Why?
Is there any collaboration between the health system and the association that serves to facilitate a first contact between a health professional and a Roma woman suffering abuse?
(IF YES) Could you describe this collaboration (health system/association)? And how would you describe the relationship between the two?
(IF NO) Do you think such a collaboration is necessary (health system/association)? Why?
In general, what are the principal characteristics needed by workers attending to these cases of violence in Roma women?
With respect to cases of abuse, what do you think is the general response to cases of intimate partner violence? What about cases of Romani women who suffer from this violence?
In general, what improvement is needed in these cases? In the primary care setting, what do you think is most needed by health professionals so that they can properly attend to Roma women who suffer violence?

The interviews lasted between 35 and 90 minutes and were conducted in Spanish. The native language of all of the participants was Spanish. Participants were interviewed within the setting of Roma associations. We continued to conduct interviews until data saturation was achieved, meaning that no new information related to the project aim was emerging. In keeping with the principles of the Declaration of Helsinki and the Belmont Report, the purpose and procedure of the study were explained, an opportunity to ask questions was provided, and written informed consent was obtained from each participant prior to data collection. Participants were informed that they could interrupt their participation at any time during the interviews. Ethical approval was sought from the Ethics Committee of the University of Alicante.

### Data analysis

The interviews were digitally recorded and then transcribed verbatim. We followed the six steps of thematic analysis as described by Braun and Clarke [30]. Each author carried out steps one and two on their own; these steps involved familiarizing ourselves with the data and generating initial codes. For step three, we compared initial codes and searched for potential themes together. Author 3 then returned to the transcripts to organize all relevant data within the potential themes. All four authors met in person and worked electronically to carry out steps four through six together, including reviewing themes, defining and naming themes and producing the report. Five preliminary categories emerged during this process, and they were condensed into the three final themes described in the Results section (Table 2).

**Table 2.** Themes, categories and main codes generated from the interviews with women from Roma or pro-Roma associations in Spain, 2014–2015.

Themes	Between persistence and rupture of restrictive gender norms			Invisibility and normalization of Violence Against Women (VAW) in the Roma community		Willingness and trust in daily work with women
**Categories**	Social construction of Romani women’s gender identity	Generational ruptures	Social changes in the Roma community. Are they real or apparent?	Delinking ethnicity from experiences with VAW among Romani women	Signs of particular/unique experiences of VAW among Romani women	Need for specific VAW measures within a general intervention model
**Selected codes**	– Elements of multiple discrimination: for being a woman, Roma, low education level and low income, among others. – Persistence of traditional notions about being ‘a Roma woman’ linked to cultural identity. – Consequences of traditional gender norms for women’s health and wellbeing	– Access to education and employment as elements that emancipate women. – Empowered youth. – Conflicts of identity. – Transition of models of femininity. – Examples of the rupture in traditional roles and their consequences	– Women who lead processes of change are also sanctioned by the community and by men. – The mirage of equality	– Invisibility as a way to avoid further damaging the image of the Roma community. – VAW is similar between Romani and non-Romani women. – VAW normalization among Roma. Real or an excuse?	– Romani women face greater obstacles – Extended family is key to understand how women make decisions – The importance of informal help-seeking resources	– From a model of information and support to a model of intervention and social empowerment. – Working to prevent VAW in a subtly ‘hidden’ way. – Personal empathy and implication of association representatives

## Results

Our analysis resulted in three themes that captured different struggles that women from Roma organizations in Spain faced in relation to gender norms, VAW and strategies for confronting VAW: ‘between persistence and rupture of restrictive gender norms’, ‘invisibility and normalization of VAW’ and ‘willingness and trust in daily work with women’.

### Between persistence and rupture of restrictive gender norms

The first struggle that informants negotiated was related to continuity and change in restrictive gender norms for Roma women. In general, the interviewees perceived that Roma women faced various forms of oppression and discrimination on a daily basis. These forms of discrimination stem from Spanish society in general, the Roma community in particular and even their own families. Being aware of the reality faced by Roma women was considered key to working with them, as the following quote illustrates:

…I think it is important to recognize the Romani woman’s role – and the behaviors that generate a man’s control of a woman – because one can understand how to work with these women… (Asociación Gitana (AG), 10)

According to interviewees, Romani women are subjected to ideal notions of femininity that emphasize motherhood and being a faithful wife, and that prioritize the needs of the family over women’s own needs. The interviews also revealed that these notions of femininity have also been present in the past in Spanish society:

The problem with the Roma population is that we mirror the majority society, but we are 100 years behind… In the machista Spanish society of the past, no Roma women who smoked were frowned upon. Towns’ women in mourning used to cover themselves. Even today, Roma women continue to do this. (AG, 6)

The importance of marriage and the fact that Roma women begin reproducing at an early age (child mothers) can collide with women’s life aspirations related to education, labor and economic emancipation etc.:

There is the role of the adult woman (related to sexual life, being a mother, taking care of the household) and the role of the child that is beneath, and dependent on (in this order), her husband (who is beneath his parents), her in-laws and her parents. (AG, 10)

The interviewees understood these gender norms as hindering women’s capacity for decision-making, empowerment and freedom, in addition to having consequences for women’s mental health:

There is a very important part of Romani women who live in a cage all of our lives without really being able to do what we want or what our body tells us; […] and you can’t do what you choose, your life doesn’t belong to you, nor your person, and in the long run this affects you psychically. (AG, 1)

However, some of the interviewed women highlighted the existence of changes in gender relations, and they even included themselves as protagonists in processes of change and personal empowerment. This is especially the case for younger women, although it is also present in the discourse of some older informants:

Now I don’t care at all what they think of me and what they say, even in my home, it doesn’t matter, my life is my own, I only have one life. But I didn’t realize this until very recently, OK? But I do want to communicate this message to other young women, and I am in a hurry to do things and try to change things in the place where I am and in the way that I can. (AG, 1)

In my 43 years I have learned that I shouldn’t stop doing what I want […] In my culture I have respect for everyone, but if I light up a cigarette here in front of an 80-year-old Roma man, he can look at me the way he wants, because I am free and I decide. (A.G, 6)

The rupture with restrictive gender norms was not seen as problem-free. In fact, it can be a difficult process due to cultural socialization and social pressure, and it can even generate a certain sense of false rupture:

That darn subconscious and the education they have given you… you have learned it from childhood and it is rooted in you… It is very difficult to break with this, maybe impossible, one tries, makes an effort, but at some point it comes out. (AG, 2)

…there are many people […] Romani and non-Romani who think they are free, but they aren’t, they live in a glass vase OK? […] They are like helium balloons that rise, but when they rise you can pull on their string and pull them back down. (AG, 6)

Capacity-building, labor market participation and social support from associations were considered key elements for producing substantial changes. With these necessary elements, a hopeful eye was cast again on younger women:

The outcome that I see is that boys and girls don’t drop out of their studies. I think that education is the starting point… Because they see the change in what they want to change. (AG, 4)

### Invisibility and normalization of VAW in the Romani community

The second struggle that informants negotiated was related to the magnitude and perception of VAW within the Roma community and in comparison with the general population. Two main elements of this struggle were raised: attempting to prevent further negative stereotypes being directed against Roma people, while at the same time acknowledging the specific characteristics of VAW when the victims are Romani women.

VAW, its consequences and victims seem to be invisible, even for Roma organizations. This fact tended to be interpreted by the interviewees as a strategy to avoid further damaging the image of the Roma community. In this way, one of the interviewees explained an experience with a report produced by her organization that wasn’t published in the end:

There was the knowledge that the rest of the Roma entities would take over because it was a way to say, ‘Well, we’re going to add more fuel to this fire and to these stereotypes that people have about the Roma community.’ (AG, 8)

Although sharing this common agreement around the invisibility and silence surrounding VAW, some informants highlighted that the situation is similar for the non-Roma population. In general, these interviewees considered that the magnitude and risk of VAW in the Roma community are probably similar to those registered among the general population. This could be interpreted as an effort to delink their ethnicity and its cultural elements from the possibility of a greater risk of abuse in Roma couples:

Just as there are people in the culture who understand a woman’s role to be a certain way, well there are also people in the Spanish culture who still understand that a woman has to stay at home, and that is the way it is. So, well, of course, there are people who understand it that way, but I don’t think that it’s the Roma culture, the Roma culture is something else […] the risk factor is being a woman and the concept of Roma culture […] is advancing more and more. (AG, 5)

However, interviewees coincided in signaling the greater obstacles faced by Romani women in comparison with the general population in being able to escape from VAW situations. Specifically, they emphasized the contradictory role that can be played by members of the community and the family, taking into account the importance of the extended family within the Roma community:

When a Roma woman marries, well, that is where there are many more problems. Because the Roma woman, in principle, becomes part of the other clan, OK, a part of her husband’s family, OK? Then, if she separates, OK?, two things happen: one is that she should leave her children, OK? […] The children become part of her husband’s family […] Later if they go with their mother, it is considered a dishonor. (AG, 7)

If this type of problem is painful and complicated for a woman, for a Roma woman it is much more so, since given that she moves in relationship to the extensive family, the problem complicates very much the family relationship, because her husband, besides being her husband, is a close family member (cousin). (AG, 9)

Another related topic that emerged in the interviews relates to the supposed normalization of the violence, and specifically VAW, within the Roma community. Some informants seemed to acknowledge the existence of this normalization when they spoke as members of their community and as women that have suffered themselves the consequences of a differential socialization related to gender:

I think we are not conscious of the fact that we are giving it power with the words that become recorded in the subconscious, ‘you have to bear it because he is your husband… It is normal that he gives you a smack. Ah! Well we have all had to bear it honey.’ These phrases that form part of daily life penetrate so deeply that when it´s your turn to go through the situation you look back and you say, ‘no, well this is normal’, you think, ‘ah! Well if my cousin’s husband hits her and there is no problem, then I will have to bear it.’ And at the end it becomes something normal. (AG, 2)

Furthermore, some of the informants considered that the opinion that VAW is more tolerated within the Roma people could affect formal responses related to detection and attention from agencies, organizations and professionals outside the Roma community:

Because if you think about it right now, here in the street [a boy insults a girl] and everyone joins in. [But if] they´re gypsies, […] everyone leaves. Because ‘no, they see it as normal, they’ve normalized it’. (AG, 6)

However, some interviewees directly rejected the existence of a normalization of VAW inside the Roma community. Actually, they pointed out the presence of informal strategies to deal with situations of violence, and specifically VAW. These strategies usually involve the implication of extended family members and/or people with authority within the community.

### Willingness and trust in daily work with women

Finally, the third struggle that informants negotiated was related to developing strategies to address VAW in their daily work with Roma women. Interviewees highlighted that strategies don’t need to be different from those that exist for all women, but should be improved in terms of quality and response capacity:

We don’t need any specific resource for Roma women […], all […] we need is that the resources we have are of good quality and are well coordinated. (AG, 7)

However, during the interviews, informants mentioned that they themselves needed to develop specific and culturally sensitive strategies that approach VAW in an indirect way, formulating the problem in a diffuse manner:

it’s a subtle question, we work on these issues but mask it; it’s what the colleagues from Valencia told you, that in the Roma women’s violence group they don’t call it that… (AG, 3)

They perceived that the Roma community generally rejects reporting crimes to the police, thereby making it difficult to develop VAW prevention strategies that focus on law enforcement and legal mechanisms:

It is unthinkable that a woman denounces her husband […], not just because of her husband but because of the whole environment, and even those who aren’t family members would say ‘What type of woman would go and denounce her husband?’, even if her eyes are like this [gesture of a hand and swollen eye], she shouldn’t denounce him. (AG, 1)

Denouncing him is impossible, they don’t denounce them, and the protocol that requires [them to denounce] in order to get out of the situation she is in, this doesn’t serve Roma women. […] You have to create strategies among women who have worked with Roma women, create strategies to help them have the power to say, ‘help me because I am in this situation’, otherwise they won’t do it. (AG, 6)

Sometimes interviewees depicted carrying out VAW prevention strategies more in response to their own sensitivity and willingness to effect change than to the ultimate goals of their organizations. It can even be concluded that VAW prevention strategies were their own initiative rather than the directives of their organizations. Consequently, the personal commitment of the interviewees combined with a strong awareness of the phenomenon and its impact on the lives of women and families was critical for the development of VAW prevention, detection and treatment:

Yes, we have worked in the area of equality, and transversally I pushed to make them take on the topic of gender violence because I think it is fundamental. (AG, 11)

Before it was something hidden, and now they just express it, right? So this causes you to become more conscious of it, right? Because sometimes it’s like, if you don’t know you can’t help at all because they don’t tell you. Unless you see something… [then] you can talk and tell her ‘when you want to, take my phone number and call me’. (AG, 11)

In this sense, the informants highlighted the role that they play individually and their organizations play in building trust with community members, including affected women, aggressors and family members:

Roma women need their time; they need trust to be able to speak about it. […] The woman who suffers, she doesn’t tell just anyone, it has to be someone of extreme confidence, that knows; they have to know that you aren’t going to turn anyone in, that it’s going to stay between you and her, and you can’t always find someone like that among the professionals or the family members. (AG, 1)

Finally, the elements mentioned put the interviewees into the contradictory situation of having to approach the topic mostly with the same resources that exist for VAW in general, but with the knowledge that the point of departure is different.

## Discussion

Cultural traditions, taboo and invisibility, willingness and trust are present in the discourses of the interviewees in relation to their experiences working with Roma women dealing with situations of violence. All of these factors give rise to tension and conflict. The activists constantly move between respect and rupture of the dominant gender norms; invisibility of VAW and the desire not to further damage the image of their community; the willingness to help the women but also the awareness that they need (and sometimes lack) institutional support; and knowledge of the ways in which informal networks can generate responses to VAW, although they can also hinder the rupture with the abuser.

In Roma families, women and men’s roles are strictly defined by patriarchal norms [25]. The expectations related to being a woman and being Roma are intimately linked to one’s position in the family. The importance of marriage in addition to the fact that Roma women bear children at a young age (child mothers) can contrast with life aspirations in terms of education, the labor market and economic independence. The majority of the women we interviewed (all of them Roma women involved in civil society organizations) have tried to break with this stereotypical image, in greater or lesser measure, reclaiming an active role in denouncing different sources of discrimination by sex, ethnicity, social class and gender [31]. However, they often highlight the relevance of knowing, understanding and even respecting elements of the dominant gender norms when it comes time to work properly with Roma women.

References to the effects of what is called a ‘mirage of equality’ are found within this conflictive discourse, which alludes to achievements in terms of legislation, political agendas and public discourse that have yet to be extended to the daily lives of women and men [32]. In this context, the interviewees mention women born in recent decades as possible protagonists of change, but who, at the same time, live with different forms of inequalities and sexism [33]. Furthermore, the real possibilities of breaking with one’s traditional position can be impaired by the fact that the Roma population does not generally participate fully in society [34]. Roma people in Spain suffer greater levels of poverty and unemployment and have greater difficulties in access to housing and to continuing in the educational system. Levels of racism and discrimination towards this group are high [35]. This rejection, perceived by the Roma population, is reflected in distrust towards institutions and services managed from outside of their community.

Representatives of the Roma movement continue their struggle against different forms of discrimination with a certain tendency, according to those interviewed, not to prioritize changes in gender identities, relations and violence. This generates a conflict between Roma activists and feminists, and between women’s organizations and the whole of the Roma community. The conflict is usually represented by men who defend the rights of the Roma community with little attention to gender inequalities. This rupture situates Roma women and their organizations outside of their own community [18,21]. For some authors, the disconnect between the defense of Roma community interests and the defense of Roma women is generated by a social context in which gender and ethnicity are erroneously assumed to be mutually exclusive rather than interrelated [36,37].

The invisibility of the real dimensions of VAW among Roma women is recognized by the interviewees. Some of them interpret this invisibility as a general characteristic of VAW, known as the ‘tip of the iceberg’ concept. This refers to the fact that the cases of VAW that are filed and those collected through investigations are only a part of the whole, and they are generally the more severe ones. The existence of other forms of violence remains hidden [38]. However, some interviewees consider this invisibility, in the case of Roma women, to be – to a certain point – understandable, without which VAW could become an anti-Roma tool [27]. This could be related to the explicit rejection by some of the informants of the supposed normalization of the problem in the Roma community. In any case, the interviewees try to frame the topic of VAW by putting emphasis on the barriers to access to services and on the need for improvement in social services (education, employment, income, empowerment, and youth and women), avoiding the criminalization of the community and Roma men [18].

Willingness or personal motivation has been identified in other contexts (for example, primary health care) as a key factor in generation of responses to VAW [39]. This motivation could go hand in hand with training, awareness of issues of gender and/or – as our interviewees argue – the experience of having worked with women in this situation. Despite the potential of this willingness, the interviewees claim the need for greater support and commitment on the part of their own organizations and other institutions. In consonance with the informants, for Wasileskia and Miller [40], success in the elimination of interpersonal violence within Roma families is linked to the application of policies that increase the quality and accessibility of social services as well as improvement in the social position of Roma families. However, these policies will not be sufficient unless Roma women are directly implicated by encouraging them to become links between their communities and different resources.

Certain study limitations should be considered in interpretation of the results. The principal limitation is that we have analyzed a series of topics that were not initially included in the interview guide. This could have conditioned the information collected and resulted in a lack of depth in some of the topics discussed. Another limitation is that the interviewers were not Roma women, although one of them was linked to an organized Roma movement. Finally, it is worth commenting that the interviewees were linked to different types of associations (Roma, pro-Roma, feminist and Roma women’s associations). This is a limitation that could influence the saturation of the discussed topics, although it is also a strength given the need to collect the opinions and experiences of a wide range of Roma organizations.

We applied the criteria described by Lincoln and Guba to enhance trustworthiness in qualitative research [41]. Transferability was enhanced by selecting participants’ profiles based on their ability to contribute to the research question. Triangulation of researchers from different disciplines and with different levels of familiarity with the setting was also used to enhance credibility. We also made an effort to contextualize the results in order to help readers evaluate to what extent our results might be applicable to other similar settings. To enhance dependability, the study adopted an emergent design throughout the research process, which contributed to making Roma women’s opinions and experiences in their dealing with VAW cases more visible. We also responded to constant change even when that implied modifications to the planned schedule or the interview guides. Quotations were also used to enhance confirmability. In order to stay closer to the text, the original Spanish version was used for coding, and translation into English only took place when categories and themes had emerged.

## Conclusions

This paper contributes to an underdeveloped topic: the analysis of Roma women, not as victims, but as protagonists of social change. Our results show the presence of numerous conflicts related to their intersectional position along different axes of discrimination. These conflicts were grouped into the following blocks: ‘between persistence and rupture of restrictive gender norms’, ‘invisibility and normalization of VAW’ and ‘willingness and trust in daily work with women’. The work carried out by a large part of the interviewees related to VAW responds more to their own initiatives and personal abilities than to strategies adopted by the organizations they are part of. It is worth highlighting that VAW, which underpins gender inequalities, seems not to form part of the fundamental core priorities of Roma and pro-Roma organizations. Finally, the results show the need to support the initiatives of Romani women as well as their own identity struggles, which are what make them true promoters of social change.

## Paper context

Previous studies about intimate partner violence (IPV) among Romani women and girls are scarce. However, the few which have been conducted coincide in indicating the importance of generating social innovations to cope with IPV among minority women’s groups such as Romani women in Spain. In this manuscript, we aim to identify different struggles that women from Roma organizations in Spain have dealt with in relation to VAW and their contacts with affected women. In order to analyze that, an intersectional approach was considered and for this reason, we consider that our results may enrich this cluster of papers about gender and its intersection with other axes of health inequalities.

## References

[1] Ellsberg M, Arango DJ, Morton M, et al Prevention of violence against women and girls: what does the evidence say? Lancet. 2015 4 18;385:1555–10. 25467575 10.1016/S0140-6736(14)61703-7

[2] Michau L, Horn J, Bank A, et al Prevention of violence against women and girls: lessons from practice. Lancet. 2015 4 25;385:1672–1684. 25467577 10.1016/S0140-6736(14)61797-9

[3] European Commission An EU framework for national Roma integration strategies up to 2020. Brussels: European Commision; 2011.

[4] Kosa Z, Szeles G, Kardos L, et al A comparative health survey of the inhabitants of Roma settlements in Hungary. Am J Public Health. 2007 5;97:853–859. 17395845 10.2105/AJPH.2005.072173 PMC1854867

[5] La Parra D, Gil-González D, Jiménez A. Los procesos de exclusión social y la salud del pueblo gitano en España. Gaceta Sanitaria. 2013;27:385–386. 23768562 10.1016/j.gaceta.2013.05.001

[6] Cook B, Wayne GF, Valentine A, et al Revisiting the evidence on health and health care disparities among the Roma: a systematic review 2003–2012. Int J Public Health. 2013;58:885–911. 24096986 10.1007/s00038-013-0518-6

[7] Vozarova De Courten B, De Courten M, Hanson RL, et al Higher prevalence of type 2 diabetes, metabolic syndrome and cardiovascular diseases in gypsies than in non-gypsies in Slovakia. Diabetes Res Clin Pract. 2003;62:95–103. 14581146 10.1016/s0168-8227(03)00162-1

[8] Parry G, Van Cleemput P, Peters J, et al Health status of gypsies and travellers in England. J Epidemiol Community Health. 2007 3 1;61:198–204. 17325395 10.1136/jech.2006.045997 PMC2652907

[9] Hassler S, Eklund L Sense of coherence and self-reported health among Roma people in Sweden - a pilot study. Int J Circumpolar Health. 2012; 71(0):1–6. 10.3402/ijch.v71i0.18438 PMC3417686 22584516

[10] Laparra M, Arza J, Fernández A, et al Diagnóstico social de la comunidad gitana en España. Un análisis contrastado de la Encuesta del CIS a Hogares de Población Gitana 2007. Madrid: Ministerio de Sanidad, Política Social e Igualdad; 2011.

[11] La Parra D Hacia la equidad en salud: estudio comparativo de las encuestas nacionales de salud a población gitana y población general de España, 2006. Madrid: Ministerio de Sanidad y Política Social, y Fundación Secretariado Gitano; 2009.

[12] Danova-Russinova S Ambulance not on the way: the disgrace of health care for roma in Europe. Report No.: 9638695536. Budapest: European Roma Rights Centre; 2006.

[13] Council of Europe. International conference of Roma Women in Europe portal webpage Strasbourg: Council of Europe. 2016 Available from: http://www.coe.int/en/web/portal/roma-women

[14] European Monitoring Centre on Racism and Xenophobia Breaking the barriers - Romani women and access to public health care. Luxembourg: Council of Europe; 2003.

[15] Prava za sve and ICVA Sarajevo Roma women for life without violence. Response of institutions to domestic violence project. Sarajevo: Rights for all. Inicijativa i civilna akcija; 2011.

[16] Sordé Martí T, Munté A, Contreras A, et al Immigrant and native Romani women in Spain: building alliances and developing shared strategies. J Ethn Migr Stud. 2012;38:1233–1249.

[17] Tokuç B, Ekuklu G, Avcioğlu S Domestic Violence Against Married Women in Edirne. J Interpers Violence. 2010 5 1;25:832–847. 19587297 10.1177/0886260509336960

[18] Izsák R The European Romani women’s movement: the struggle for human rights. Development. 2009;52:200–207.

[19] Oprea A Romani feminism in reactionary times. Signs: J Women Cult Soc. 2012;38:11–21.

[20] Sandu A Participation, representation and voice in the fight against gender violence: the case of the women’s movement in Spain. J Int Women’s Stud. 2013;14:24–39.

[21] Schneeweis A Empowered leaders and alone in community: stories of Romanian Roma health mediators. Women’s Stud Commun. 2013;36:167–188.

[22] Springer KW, Hankivsky O, Bates LM Gender and health: relational, intersectional, and biosocial approaches. Soc Sci Med. 2012;74:1661–1666. 22497844 10.1016/j.socscimed.2012.03.001

[23] Bauer GR Incorporating intersectionality theory into population health research methodology: challenges and the potential to advance health equity. Soc Sci Med. 2014 6;110:10–17. 24704889 10.1016/j.socscimed.2014.03.022

[24] Hankivsky O Women’s health, men’s health, and gender and health: implications of intersectionality. Soc Sci Med. 2012;74:1712–1720. 22361090 10.1016/j.socscimed.2011.11.029

[25] Ceneda S Romani women from central and Eastern Europe: a ‘Fourth World’, or experience of multiple discrimination. London: Refugee Women’s Resource Project. Asylum Aid; 2002.

[26] Corsi M, Crepaldi C Ethnic minority and Roma women in Europe: a case for gender equality: synthesis report. Report No.: 9279129813. Brussels: Publications office of the European Union; 2010.

[27] Jovanovic J, Kocze A, Balogh L Intersections of gender, ethnicity, and Class: history and future of the Romani women’s movement. FES Working Papers. Budapest: Friedrich-Ebert-Stiftung and CEU Center for Policy Studies; 2015 p. 16.

[28] Gobbo F Cultural intersections: the life story of a Roma cultural mediator. Eur Educ Res J. 2004;3:626–641.

[29] Schneeweis A Power, gender, and ethnic spaces: geographies of power in Roma communities. J Commun Inq. 2015 9 28;2015:88–105.

[30] Braun V, Clarke V Using thematic analysis in psychology. Qual Res Psychol. 2006;3:77–101.

[31] Schultz DL Translating intersectionality theory into practice: a tale of Romani-Gadže feminist alliance. Signs: J Women Cult Soc. 2012;38:37–43.

[32] García Prince E El espejismo de la igualdad: el peso de las mujeres y de lo femenino en las iniciativas de cambio institucional. Otras Miradas. 2006;6:24–30.

[33] Rodríguez MES Hijas de la igualdad, herederas de injusticias. Madrid: Narcea Ediciones; 2008.

[34] Koutská T, Kajanová A Domestic violence from the perspective of Roma women. In 1st international e-conference on optimization, education and data mining in science, engineering and risk management 2011.

[35] La Parra Casado D, Gil Gonzalez D, De La Torre Esteve M The social class gradient in health in Spain and the health status of the Spanish Roma. Ethn Health. 2016 10;21:468–479. 26458079 10.1080/13557858.2015.1093096

[36] Oprea A The arranged marriage of Ana Maria Cioaba, intra-community oppression and Romani feminist ideals: transcending the ‘primitive culture’ argument. Eur J Women’s Stud. 2005;12:133–148.

[37] Sokoloff NJ, Dupont I Domestic violence at the intersections of race, class, and gender: challenges and contributions to understanding violence against marginalized women in diverse communities. Violence Against Women. 2005;11:38–64. 16043540 10.1177/1077801204271476

[38] Vives C, Álvarez-Dardet C, Caballero P Violencia del compañero íntimo en España. Gaceta Sanitaria. 2003;17:268–274. 12975049 10.1016/s0213-9111(03)71746-4

[39] Goicolea I, Hurtig AK, San Sebastian M, et al, Developing a programme theory to explain how primary health care teams learn to respond to intimate partner violence: a realist case-study. BMC Health Serv Res. 2015;15:228. 26054758 10.1186/s12913-015-0899-8 PMC4460973

[40] Wasileski G, Miller SL “Bad” victims?: understanding social service providers’ responses to Roma battered women. Int J Comp Appl Crim Justice. 2014;38:173–189.

[41] Lincoln YS, Guba EG Naturalistic inquiry. Newbury Park, CA: Sage; 1985.

